# The rate of leukocyte telomere shortening predicts mortality from cardiovascular disease in elderly men

**DOI:** 10.18632/aging.100007

**Published:** 2008-12-04

**Authors:** Elissa S. Epel, Sharon Stein Merkin, Richard Cawthon, Elizabeth H. Blackburn, Nancy E. Adler, Mark J. Pletcher, Teresa E. Seeman

**Affiliations:** ^1^ University of California, San Francisco, Department of Psychiatry, San Francisco, CA, 94143, USA; ^2^ University of California, Los Angeles, David Geffen School of Medicine, Los Angeles, CA, 90095-1687, USA; ^3^ University of Utah, Human Genetics, Salt Lake City, UT 84112, USA; ^4^ University of California, San Francisco, Department of Biochemistry & Biophysics, San Francisco, CA, 94158, USA; ^5^ University of California, San Francisco, Department of Epidemiology & Biostatistics, San Francisco, CA, 94107, USA

**Keywords:** aging, longevity, telomere length, cardiovascular disease, mortality

## Abstract

Telomere length (TL) has been proposed as a marker of mitotic cell age and as a general index of human organismic aging. Short absolute leukocyte telomere length has been linked to cardiovascular-related morbidity and mortality.  Our aim was to test whether the rate of change in leukocyte TL is related to mortality in a healthy elderly cohort.  We examined a subsample of 236 randomly selected Caucasian participants from the MacArthur Health Aging Study (aged 70 to 79 years).  DNA samples from baseline and 2.5 years later were assayed for mean TL of leukocytes.  Percent change in TL was calculated as a measure of TL change (TLC).   Associations between TL and TLC with 12-year overall and cardiovascular mortality were assessed.  Over the 2.5 year period, 46% of the study participants showed maintenance of mean bulk TL, whereas 30% showed telomere shortening, and, unexpectedly, 24% showed telomere lengthening. For women, short baseline TL was related to greater mortality from cardiovascular disease (OR = 2.3; 95% CI: 1.0 - 5.3).  For men, TLC (specifically shortening), but not baseline TL, was related to greater cardiovascular mortality, OR = 3.0 (95% CI: 1.1 - 8.2).  This is the first demonstration that rate of telomere length change (TLC) predicts mortality and thus may be a useful prognostic factor for longevity.

## Introduction

Understanding the aging process is central to preventing age-related disease burden and premature mortality. Many different measures have been suggested as having prognostic value for mortality.  Cellular aging may offer insights into organismic aging relevant to diseases of aging such as CVD.  Telomeres, the protective nucleoprotein structures capping the ends of eukaryotic chromosomes, can serve as markers of mitotic cell age and  replicative  potential.  With  every  cell  division, a portion of the telomere cap is not replicated due to the "end replication problem" - that is, DNA polymerase does not completely replicate the end of a DNA strand [1].  Hence, cells in certain older organisms, including humans, have shorter telomeres on average than cells in younger organisms.

Telomere length change (TLC) depends on many factors, prominent among them the rate of cell divisions and level of telomerase, a cellular ribonucleoprotein reverse transcriptase enzyme that replenishes telomeric DNA and thus lengthens the telomere.  In cells lacking sufficient levels of telomerase, telomeres progressively shorten with successive cell divisions.  If the telomere shortening represents a clock ticking forward on cells' lifespans, telomerase can slow or reverse this clock [2], making the two an intricately interdependent dynamic system. Indeed, in vitro studies show that telomeres can lengthen - activated B cell telomere length increases as these cells multiply in germinal centers in response to pathogenic challenge [3]. TLC in part reflects the balance between telomere elongation by telomerase action versus telomere shortening processes.

Cellular senescence may underlie the progression of diseases associated with organismic aging [4].  Mice bred without telomerase develop shorter telomeres, and show premature aging, including hair graying, impaired wound healing, reduced proliferation of lymphocytes, and, in later generations, early mortality and infertility [5].  Humans with a rare genetic disorder (dyskeratosis congenita) that leads to half the effective gene dosage of telomerase show early mortality and increased incidences of fibrosis, cancer, progressive bone marrow failure and other indications of premature aging, and other premature aging syndromes are also often characterized by shortened telomeres [4,6-8]. Despite these lines of evidence, among the general population of healthy humans without pathologic premature aging syndromes, little direct data exist to link cellular aging with organismic aging.

The strongest evidence that cellular aging, as reflected by shorter telomeres, might be associated with organismic aging has until now been derived from cross sectional studies. Shorter telomere length (TL) in leukocytes has been associated cross-sectionally with CVD and its risk factors, including pulse pressure [9-11], obesity [12,13], vascular dementia [14], diabetes [13,15,16], CAD [17], and myocardial infarction [18] although not in all studies [19].   TL has also been shown to predict CVD events (MI and stroke) in men under 73 years old [20]. Cawthon and colleagues found that TL predicted earlier mortality, particularly from CVD and infectious disease, in a sample of 143 healthy men and women 60 years and older [21]. This suggested that poor telomere maintenance may serve as a prognostic biomarker of risk of early mortality.  Since then, additional studies  have found blood TL predicts mortality, in large twin studies [22,23], and in Alzheimers [24], and stroke patients [25]. However, other reports, notably those with very elderly cohorts, have failed to find an association between TL and mortality [26].

A single TL assessment, however, leaves open the possibility that TL at birth, rather than rate of telomere attrition, accounts for this association with mortality.  One might have expected, given the low rate of attrition throughout life, that TL at birth would be a strong predictor of TL later in life. However, twin studies indicate non-genetic factors can have significant effects on telomere length later in life; telomere length was similarly related in identical compared to fraternal male twins over 70 years old, suggesting a large non-genetic influence [27], and identical twins who exercised had longer leukocyte telomeres than the identical twin who did not [28].  Further, twin studies show that telomere length predicts mortality beyond genetic influences [22,23].   Hence, longitudinal studies that examine telomere changes over time within individuals are needed to test the prognostic value of the rate of telomere length change (TLC).

In one of the only published studies of TLC over time in humans, a study of 70 adults found that a small percentage (10%) of subjects showed leukocyte telomere length maintenance or lengthening over a ten year period [12]. No studies we are aware of in humans have systematically examined TLC within a short period of only a few years, and how this may or may not be linked to subsequent mortality.  The current study examined TL and TLC in a high functioning sample of 70-79 year olds.  We aimed to: 1) Describe the natural history of telomere length change over a 2.5 year period in a sample of elderly men and women; and 2) Test TL and particularly TLC as predictors of mortality.  Lastly, we explored whether the combination of short TL and greater TLC predicted greater risk of subsequent mortality than either one indicator alone.  We report here that TLC over the next 2.5 years did indeed predict 12-year mortality from cardiovascular disease in men. Hence we propose that the rate of leukocyte telomere shortening is a potentially useful prognostic for cardiovascular disease.

## Results

### Participants

The participants were aged 70 to 79 at baseline (1988), with an average age of 73.7 years (SD = 2.87).  Their ethnicity was Caucasian (100%). The average BMI, blood pressure, alcohol intake, physical activity, and percent of smokers and of those with diabetes are shown in Table 1.  We also examined sociodemo-graphic and health variables by short and long TL groups.  As shown in Table 1, there were no group differences in any of the sociodemographic or health variables examined by long and short TL groups.

**Table 1.** Sociodemographic and health status for total sample and by short and long TL groups (% or Means and Standard Deviations). ^*^Short TL: defined as TL below the median/ Long TL: defined as TL above the median. ^a^p<0.01. There were no significant differences in these health and behavioral factors by TL group, above, or by sex and TL group (not shown).

	Total sample N = 235	Short TL* N = 117	Long TL N = 118
T/S ratio	1.1 (0.24)	0.94 (0.13) a	1.3 (0.16)
Mean Age	73.7 (2.9)	73.7 (2.9)	73.7 (2.9)
Mean Education (years)	10.5 (2.5)	10.6 (2.5)	10.4 (2.6)
Mean Diastolic BP	75.4 (10.1)	74.7 (10.0)	76.0 (10.2)
Mean Systolic BP	135.0 (17.1)	134.8 (16.5)	135.2 (17.7)
Hypertension (%)	49.4	47.9	50.9
Diabetes (%)	20.4	22.4	18.3
Mean Physical Activity	18.9 (25.5)	21.3 (27.3)	16.4 (23.4)
Current smokers (%)	18.7	16.2	21.2
Mean Alcohol intake (ounces/month)	4.5 (11.1)	4.5 (9.9)	4.5 (12.2)

Further, we examined these factors in TL groups by sex, and still found no significant differences across the groups (men with long vs. short TL, and women with long vs. short TL).

### Natural history of Telomere Length Change (TLC) over 2.5 years

The average baseline TL was 1.1 t/s (4697 base pairs or bp), and ranged from 0.46 to 1.9 t/s.  Consistent with other studies, women had longer TL at baseline (mean t/s 1.17; SD = .233), compared to men (mean t/s 1.09; SD = .233, p < .008). Hence, when TL was divided into long and short, based on a median split for the entire sample of men plus women, there tended to be  more women in the long telomere group (58.3% women), and more men in the short telomere length group (56.7% men). The mean TL at the follow-up visit 2.5 years after baseline wassimilar, (mean t/s 1.1; with a range from 0.76 to 1.8). The raw t/s change score values ranged from -.75 to .60.  This corresponds to a range from a net loss of 1067 bp/year to a net gain of 925 bp/year, at the extremes.  There was no significant gender difference in %TLC.

To quantify the extent of more substantial (and likely more meaningful) decreases or increases in TL, we categorized people based on change scores that were outside the 7% range of the variability expected for the assay.  To be conservative, we used differences of at least +/- 15% from the baseline TL value as a cut point for indicating a reliable and large change from baseline.  Participants who showed less than a 15% change (increase or decrease) from their baseline TL were categorized as TL Maintainers. TL maintainers comprised 55% of the sample.  For the purposes of this analysis, those who showed a decrease in TL of greater than 15% are described as having significant shortening, and comprised 30% of the sample.  Those with greater than 15% increase in TL are described as having significant lengthening, and were 24% of the sample.

### Predictors of %TLC

Spearman correlations with %TLC were performed for several candidate sociodemographic and self reported health behaviors. There were no consistent patterns and correlations were weak, as follows:  Age (within the narrow 70-79 year baseline age span for this cohort) was not related to TL or %TLC for women, but was related to greater %TLC for men (r = -.27, p < .05), in that older men showed greater rates of telomere attrition.  BMI was related to greater %TLC (greater decreases), in women (rho = -.25, p < .05), but not significantly for men (rho = -.12, ns).  Alcohol use was also related to greater %TLC (greater decreases), again in women only (rho = -.31, p < .05). TL and %TLC were not associated with education (rho = -.01, ns), pack years of cigarettes (rho = -.04, ns), or physical activity (rho = .07), all ns.

**Table 2.** CVD Mortality rates (%) by gender for each Predictor. ^*^p < 0.05 difference within sex groups

		**Men**	**Women**
Baseline TL	Short	25.8%	29.4% *
	Long	24.0%	** 13.2%**
TL Change	Shortened	46.7% *	16.7%
	Maintained	** 17.7%**	18.4%

### TL and %TLC predict mortality?

**Figure 1.**
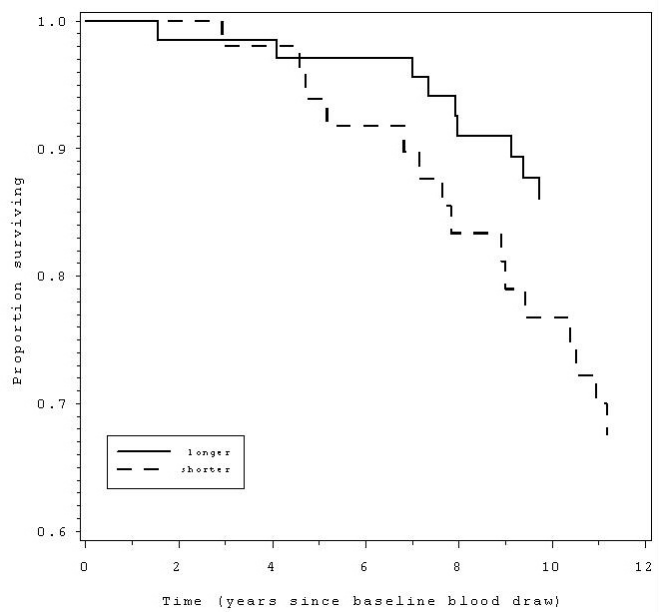
Those with shorter (below median) telomere length at baseline (dashed line) had 2.3 times greater likelihood of mortality over the following 12 years compared to those with longer telomeres (solid line).

By 2000 (12 years from the beginning of the study), 102 (43.4%) participants were known to have died, according to death certificates (42 women, 60 men). There were no associations of TL or %TLC on overall 12 year mortality. We then examined mortality from different causes in relation to leukocyte TL or telomere length change. There were not enough deaths due to infectious disease (n = 6) to examine independently. More than half of deaths (53) were from cardiovascular disease (24 women, 29 men), the main outcome in this study.   CVD Mortality rates by gender for baseline TL and %TLC are listed in Table 2.   For the sample as a whole (men and women combined), baseline TL weakly predicted CVD mortality, a relationship which achieved only marginal statistical significance, p < .10. This trend is consistent with Cawthon's previous study (2003).  However, when we examined the sample by gender, women with shorter baseline TL were 2.3 times more likely to die from CVD over the next 12 years compared with those with longer baseline TL (95% CI = 1.0 - 5.3, p < 0.05, Table 3, Figure 1).  Specifically, 20% of women had died from CVD, and of these, the majority (62.5%) were in the short TL group.  This effect held only for women, with no association of TL with CVD mortality for men (p = .60).  Although not significant, Cawthon et al (2003) also found a marginally stronger effect of TL on mortality for women as compared with men.

**Figure 2.**
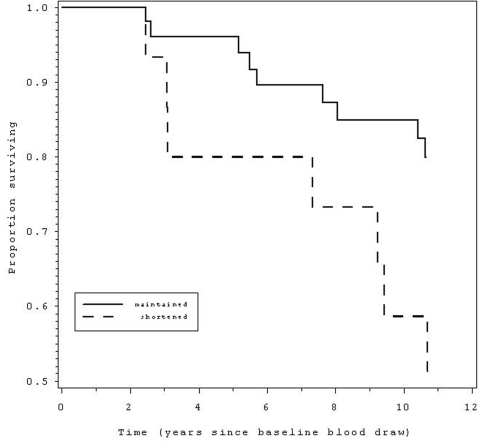
Those with telomere shortening over a 2.5 year period (dashed line) had 3.0 times greater likelihood ofmortality over the 12 years since the baseline blood draw, compared to those without telomere shortening (solid line).

We next examined rate of telomere shortening (%TLC), categorically, in relation to subsequent mortality, comparing those in the lowest quartile of %TLC (representing those with the greatest shortening) to the rest of the sample.  For women, there was no association of 12 year CVD mortality with the rate of TL shortening during the 2.5 years monitored at the beginning of the 12 year period (p = .98).  Strikingly, TL shortening rate in the men was linked to greater CVD mortality (hazard ratio = 3.0, 95% CI = 1.1 - 8.2, p < .04, Table 3, Figure 2).

A final set of secondary analyses examined the combined impact of having both shorter baseline TL and experiencing a decline in TL over time on CVD mortality and overall mortality. Given the strong relation between baseline TL and change in TL, with greater shortening seen in those with longer rather than shorter baseline TL, there were insufficient numbers of participants who had both short TL and shortening over time when examining CVD mortality. When examining overall mortality, there were nine participants in the both short baseline and shortening over time category.  A Chi Square testing baseline TL (long vs. short) by change (shortening vs. no shortening) by mortality was significant, X2 (3) = 8.70, p < .03. The men with short baseline TL and maintenance or lengthening over time were more likely to be alive 12 years later (20 of 29, 69%), compared to those with short baseline TL and telomere shortening (1 of 8, 13%).    In contrast, for men with relatively long baseline telomeres, there was no apparent effect of rate of shortening on mortality: those with telomere lengthening over time tended to be equally likely to be alive 12 years later (40%), compared to those with telomere shortening (58%).

## Discussion

Telomere maintenance has emerged as a significant determinant of the ability of mitotic cells to continue proliferating [29]. Cawthon et al. (2003) have previously shown in a cross sectional study that telomere length in late human adulthood can predict longevity.  Here we extend and replicate these findings: we  found that in older women, short telomere length (below average baseline TL) was associated with almost three times the risk of 12-year mortality from cardiovascular disease compared to women with longer baseline TL.  Further, we report a novel association of failure to maintain telomere length with cardiovascular disease mortality; we found that men who showed leukocyte telomere shortening over the short period of 2.5 years were subsequently three times more likely to die from heart disease than those who maintained leukocyte telomere length. Exploratory analyses also found that among men, those with both shorter baseline TL and shortening over the 2.5 year interval (though only a small subset of the total sample) had extremely high 12-year mortality (87%), as compared to men who also had short baseline telomeres, but who showed stable maintenance or lengthening of their telomeres during this period (31%).  Given the small sample size this secondary analysis must be replicated.

What factors might lead to faster telomere shortening? Telomere shortening, especially in the face of already short telomeres, is indicative of insufficient telomerase, as cells with short telomeres, but with adequate telomerase, can maintain proliferation and longevity [5,30].  Thus, it is in part the co-occurrence of short telomeres and low telomerase activity that appears to increase the risk of cell death in vitro [31].  More specifically, in relation to CVD, telomerase is crucial for healthy cardiovascular cell functioning [32], and has been linked to cardiovascular disease risk factors in vivo [33].  We speculate that low telomerase, as indicated by the rate of telomere shortening, may also have contributed to the more rapid decline in cardiovascular health and subsequent earlier mortality observed in men.

Another interesting finding was that in general, people in this study with short leukocyte telomeres tended to have a slower rate of telomere shortening over time, compared to those with longer telomeres.  This relation between telomere length and change in telomere length was strong (r = -.71).  Such a finding is consistent with the available information about telomerase action and the consequences of telomere shortening in cells, in which telomerase preferentially elongates shorter rather than the longer telomeres [31,34], and cells with critically short telomeres become underrepresented in the cell population because they cease to proliferate. This inverse relationship also underscores the potential importance of adjusting for baseline telomere length when examining rate of change, since we found the two are strongly inversely related.  One untested possibility for this inverse relationship is that people with short telomeres may have upregulated telomerase, which would lead to less attrition per replication, and thus prevent loss of telomere length over years.  However, there is likely to be strong selection for those cells in vivo that have maintained telomeres above critically short lengths, and thus there may be an in vivo selection for cells with short telomeres that also have higher levels of telomerase.  These are all salient questions for future research.

It is notable that cross-sectional TL did predict mortality in this sample of women, given their older age (70 to 79 years old).  This is consistent with two population-based twin studies examining cross-sectional telomere length in people of this age range or older [22,23]  but discrepant with four studies, which have found weaker [21] or no [26,35,36] effects for mortality in participants over 70 years old. The present study used a subset of participants from the MacArthur Study of Successful Aging, which only enrolled participants with good cognitive and physical functioning.  In this respect, it is an atypical sample of elderly people, who are possibly biologically younger than the unselected elderly samples typically studied. This may explain why TL served as a predictor in this elderly sample but not in other elderly samples.   Further, selection bias for healthy elderly men in the present study may in part account for why cross-sectional TL in men did not predict mortality in men, as it did in women.  Men who are very healthy at 70 to 80 years old (as in this study) are likely even more highly selected than the women given the higher mortality rates for men [37].  Thus they may be more selected for having some underlying resiliency toward age-related diseases than would be true for women.

The mechanism by which peripheral blood leukocyte telomere shortening may be linked to mortality is not yet understood.  Leukocyte telomere length may serve as a proxy for whole body aging or cardiovascular aging, because leukocyte telomere length has been correlated with telomere length in other tissues [14,38].  Leukocyte telomere length may also serve as a proxy for biochemical stress, such as oxidative stress and inflammation, known to both shorten telomeres and contribute to CVD [39]. Both alcohol excess and obesity are thought to create a milieu of oxidative stress [40,41]. Indeed, among women in the current study, greater obesity and alcohol use and obesity at baseline were related to greater telomere attrition rate. The finding with obesity is consistent with other studies which have found concurrent cross-sectional relationships between BMI and TL [13].  Alcohol use has not been previously linked to telomere length.

Lastly, leukocyte telomere shortening has important functional consequences that may contribute directly to the pathogenesis of CVD.  Short telomeres lead to cessation of cell division and can elicit cell death and, in the absence of fully functional cellular damage checkpoints, can lead to genomic instability via end-to-end chromosome fusions [5,6,31]. When telomeres become critically shortened in leukocytes, these cells become senescent and secrete pro-inflammatory cytokines [42,43].   Thus, a senescent immune system can contribute to a pro-inflammatory milieu, and senescent macrophages can contribute directly to atherosclerotic plaques [32].

While such mechanistic pathways are yet to be elucidated, our findings suggest that telomere rate of change may be an important predictor of human longevity.  Rate of attrition is informative in that it may reflect genetic, biological, and lifestyle (behavioral) factors.  This study suggests the possibility that telomere length changes over the short term might be a clinically useful measure of health status and risk.  However, this study is limited in that we cannot infer causality, and the findings need replication from larger samples before TLC is considered a validated predictor of mortality.

**Table 3.** Hazards Ratios and 95% CI for telomere predictors for CVD mortality, adjusted for age. ^*^p < .05 difference within sex groups

		**MEN**	**WOMEN**
**Baseline TL**	Short	1.2 (0.6-2.6)	2.3 (1.0-5.3)*
	Long	Reference	Reference
**TL Change **	Shortened	3.0 (1.1-8.2)*	1.0 (0.3-3.7)
	Maintained/+	Reference	Reference

## Materials and methods

Participants and procedures. Participants were men and women, from a population-based sample in East Boston (one site of the three-site MacArthur Study of Successful Aging).  Subjects were selected from within a larger population-based NIA-funded cohort study by a score in the upper tertile on six markers of mental and physical health, as described in detail in Berkman, Seeman et al, 1992, and all provided written informed consent.  This study received approval from an institutional review board at each site and was conducted in accordance with the 1964 Declaration of Helsinki (see The World Medical Association: The Declaration of Helsinki www.wma.net/e/policy/b3ht) and International Conference on Harmonization/Good Clinical Practice guidelines. Written informed consent was obtained from each patient before participation.

Though they had good cognitive and physical function, they could have chronic disease.  Response rates were over 90%.   Men (N=116) and women (n=120) were randomly sampled from among the approximately 800 with available baseline (1988) and follow-up (1991) DNA.   Blood was drawn at various times of day on the baseline visit, and on a follow-up visit for 134 participants, 2.5 years later (1991), including 66 men and 68 women.  Buffy coat was stored at -80^0^C.  DNA was later extracted at the UCLA General Clinical Research Center (Los Angeles), frozen, and shipped to Dr. Cawthon's laboratory (University of Utah) for TL assays (see below).

Statistical analyses. We first examined the relation between TL and TLC variables to see which measure of TLC was most independent of baseline TL.  Spearman correlation was performed between TL and %TLC (rho = -.71, p < .0001).  The correlation between TL and raw TLC (rather than % change) was similarly large  (rho =  -.66, p < .0001):  Those with longer TL at baseline showed greater rate of change (shortening), and conversely, those with shorter TL at baseline showed slower rate of change (less shortening or more lengthening). In light of this, we chose percent change (vs. a raw change score) as the measure of change independent of baseline length, because it adjusts for baseline TL, and hence percent of telomere length change (%TLC) was used in all analyses.

TL and %TLC were used as continuous variables.  To examine correlates of TL and %TLC, Spearman rank order correlations were used.  To examine whether TL and %TLC were predictors of mortality, Cox Proportional Hazards analyses were used, using survival time calculated in days. TL and %TLC were also exa-mined as categorical variables - baseline TL (short or long, based on a median split) and shortening over time (yes or no, with shortening reflecting the lowest (most negative) 25%ile of change. Lastly, given known gender differences in TL and mortality, all analyses were done across the group and also by gender.

Telomere length assay. Blood was drawn in subject's home and processed soon after. Buffy coat was stored at -80^0^C.  DNA was extracted by the UCLA GCRC, with Gentra system kits.  Telomere length was measured from DNA using a PCR-based assay as follows:   Two quantitative PCR reactions are performed in separate reactions, consisting of an amplification of telomere sequence (T), and of a specific single copy gene in the genome (S). The ratio of T/S corresponds to telomere length, when multiplied by a standardization factor.  The conversion factor from t/s to base pairs (bp) for this study is 4270.  All assays are performed in duplicate, have high reliability and validity [44], and have been used in multiple prior studies [21,24].

Telomere length, as measured by T/S ratio, was normally distributed, with a skew of 0.33 and kurtosis 0.14. TLC was calculated as both raw change (TL2 minus TL1), and a percent change   ((TL2-TL1)*100)/TL1, and categorically into TL change groups, as described below.  We excluded one subject with a change in telomere length greater than four standard deviations from the mean, making the final sample size 235.

Mortality. Twelve-year mortality data (from 1988 to 2000) were obtained from a National Death Index search that provided date and cause of death information based on death certificate data.  Data on overall mortality and CVD mortality was used, as described in Results below.

Health behaviors and indices. Smoking was measured by pack-years (average reported packs/day * years smoked).  Alcohol consumption was measured based on participants' reports of how much beer, wine and hard liquor they usually consume per month, and then converted to an average monthly quantity of ethyl alcohol consumed. Physical activity was measured as the sum of reported participation in strenuous work activities and strenuous recreational activities.  The detailed measures are described in Seeman et al, 1995 [45].  Height and weight were based on self report.  BMI was calculated as weight/height^2^ in kg/m^2^.  Blood pressure was measured as the average of two resting, seated assessments. Presence of diabetes was determined by self report.
